# Workplace Violence Against Nurses: Challenges and Solutions for Europe

**DOI:** 10.1177/15271544231182586

**Published:** 2023-07-20

**Authors:** Paul de Raeve, Andreas Xyrichis, Francesco Bolzonella, Jochen Bergs, Patricia M. Davidson

**Affiliations:** 1 619571 European Federation of Nurses Associations, Ixelles, Brussels, Belgium; 2 4616 Faculty of Nursing, Midwifery & Palliative Care, King's College London, London, UK; 3 School of Business and Economics, 5211 Maastricht University, Maastricht, Limburg, The Netherlands; 4 Faculty of Medicine and Life Sciences, Healthcare & Ethics Research Group, UHasselt – 54496 Hasselt University, Hasselt, Limburg, Belgium; 5 University of Wollongong, Dean Emerita Johns Hopkins University (US), Wollongong, New South Wales, Australia

**Keywords:** nurses, nursing care, healthcare, policy, workplace violence, resilient workforce

## Abstract

We report the results of a mapping exercise by the European Federation of Nurses (EFN) on challenges and solutions related to violence against nurses. This is an issue of growing international concern, with the problem accentuated during and following the COVID-19 pandemic. Following a cross-sectional observational design, an online questionnaire was distributed among 35 national nurses’ associations across Europe in March 2021. Face validity was achieved through an expert panel. Descriptive statistics were used for data analysis, including counts, percentages, and tabulation. Qualitative data analysis followed thematic synthesis techniques. Three main findings are noted. First, violent incidents against nurses are under-reported due to fear of victimization, employer discouragement, and the perception that reporting will not make any difference. Second, perpetrators of violent acts extend beyond patients and families to include health professionals of different ranks. Third, violent incidences have a significant adverse effect on nurses’ health and retention, leading to nurses reducing their working hours or opting for part-time work. Violence against nurses is an expression of a broader problem that is rooted in the failure to recognize and manage violence at the level of the healthcare organization, and the absence of appropriate legislation to maintain minimum standards of safe working environments. This is partly the result of inadequate European Union-wide legislation targeting workplace violence in the health professions. Nurses need more institutional support through dedicated funding aimed at targeted interventions, more legislative commitment to ratify policies against discrimination, and an opportunity to voice the needs to the appropriate policymakers with the ability to bring significant change to existing conditions. Given the severity of the situation, inaction could lead to irreplaceable damage to the nursing workforce, compounding pressures resulting from the COVID-19 pandemic. Ultimately, this situation can further drive existing nurses out of the profession, weakening health systems worldwide.

## Introduction

Violence against nurses has been shown to lead to inevitable and unpredictable harm to nurses’ careers, with many nurses experiencing significant and lasting psychological trauma (Zhang, Zheng et al., 2021). Nurses report that violent incidents are increasing and that they are forced to accept violence as part of their job (Najafi et al., 2018). The triggers of violence against nurses are complex, including such things as unmet patient/family expectations and inefficient organizational management. Violent incidents can lead to lower quality of patient care and intention to leave the nursing profession, leading to further organizational inefficiencies and negative patient experiences, triggering further incidents as part of an endless cycle of violence.

Nursing is a highly feminized profession. Women across Europe are frequently victims of violence and abuse (European Commission, 2021). Many live with the fear of expressing themselves, of being judged for the way they dress, and of being assaulted. Many of these women are nurses who, every day, work under difficult and high-risk conditions facing severe incidences including punching, kicking, biting, and scratching, as well as threats (Dafny & Beccaria, 2020). A significant incidence of violence against women take place in the workplace. A global problem, workplace violence against women has been on the rise across many European countries during and following the COVID-19 pandemic (De Raeve et al., 2021).

## Background

On International Women's Day, the European Commission Directorate General for Justice published its 2021 report on gender equality in the European Union (EU) (European Commission, 2021), which showed the negative impact of the COVID-19 pandemic on women. The pandemic exacerbated inequalities between women and men in many areas of life, rolling back progress and the hard-won achievements of recent years. Statements by senior officials exemplify the Commission's concerns: “Women are at the frontline of the pandemic, and they are more affected by it. We can't afford sliding back; we must continue to push for fairness and equality. This is why the EU has put women at the heart of recovery and obliged Member States to include gender equality in investments funded from Recovery and Resilience Facility,” said Vice-President for Values and Transparency, Věra Jourová. Moreover, Commissioner for Equality, Helena Dalli, added: “Despite the disproportionate impact on women's lives due to the COVID-19 crisis, we need to use this situation as an opportunity. We are determined to strengthen our efforts, continue progressing and not allow a backlash on all the gender equality gains made” (European Commission, 2020, 2021).

Tackling the issue through various means, the European Parliament published a Briefing on “Violence against women in the EU: State of play” (European Parliament, 2020), which referred to violence against women as a “violation of human rights and a form of gender-based discrimination, and constitutes a major obstacle to gender equality.” In addition, the Fundamental Rights Agency (FRA, 2021) released a survey on “Crime, safety, and victims” revealing the range of crimes and violence faced by women. FRA Director Michael O'Flaherty emphasized the urgency of addressing this issue, stating that the safety concerns and instances of harassment and violence experienced by women in Europe cannot be ignored: “EU countries need to step up their efforts to better support women. We need to do much more to tackle violence against women and honour their rights. And we need to do it now.”

In the survey, the FRA analyzed and reported data on harassment, violence, and reporting. The survey showed that 39% of the women were victims of harassment, and more women than men were subject to harassment of a sexual nature. About 37% of the women surveyed suffered physical violence at home. The physical and psychological damage suffered by the victims was high. FRA's previous survey on violence against women (2014) showed that in 11% of cases, the most severe incident of physical and/or sexual violence by a non-partner involved a perpetrator from the work context, such as a client, co-worker, or supervisor. Also, due to fear of being assaulted or harassed, 83% of 16–29-year-old women limit their movements and activities. About 68% of women do not report the violence they have suffered. This means that in official crime reports, violence against women is underestimated.

The World Health Organisation (2021) published the most extensive study ever conducted on this topic. It showed that one in three women globally experiences violence, with younger women among those most at risk. Dr. Tedros Adhanom Ghebreyesus, WHO Director-General, said: “Violence against women is endemic in every country and culture, causing harm to millions of women and their families, and has been exacerbated by the COVID-19 pandemic.” Many women who are victims of violence are also subject to abuse and harassment in their workplace. This is also the reality for many nurses 92% of whom are women (Al-Qadi, 2021).

The World Medical Association recently denounced the problem of workplace violence towards nurses as an international emergency that “undermines the very foundations of health systems and impacts critically on patient's health.” The problem of verbal and physical violence against nurses is an underestimated component of today's worldwide public health problem. The issue of violence against nurses is receiving inadequate attention from policymakers. At the moment, legislative efforts to resolve the problem remain insufficient.

It is known that violent incidents are more common in mental health and emergency departments, as nurses have to confront a particular segment of patients that have severe conditions and often express unpredictable behaviors of aggression (Caruso et al., 2021). Perpetrators are often socially justified because of their illness and nurses are expected to understand their abusive behavior, forgive them, and not complain. However, violence is not limited to specific hospital departments.

Based on literature reviews from the past decade, many violent incidents against nurses can be attributed to either organizational deficiencies, which increase work pressure and predispose nurses to abuse (Arnetz et al., 2018; Gümüşsoy et al., 2021; Khan et al., 2021; Zhang, Zheng et al., 2021), or to instances of power abuse perpetrated by colleagues in higher positions (Beale & Hoel, 2011; Giménez Lozano et al., 2021). Furthermore, such cases can also be regarded as a manifestation of the overall discretization of the nursing profession, coupled with persistent gender-based discrimination in the workplace (FRA, 2021; Petrogloou, 2019; Rechel et al., 2006).

The negative effects of workplace violence on nurses are significant and include low productivity, professional commitment, and increasing turnover intention (Chang et al., 2019). Studies across different settings and countries have also claimed links between violence and nurses’ low job satisfaction (Abo Gad & Elhossiny, 2013; Boafo, 2018), increase in burnout, humiliation, and stress (Kafle et al., 2022), as well as emotional exhaustion and concerns over patient safety (Kim et al., 2021).

Violence against women also incurs substantial economic burdens, as highlighted by the United Nations (UN, 2016). For instance, intimate partner violence in the United States of America (USA) amounts to annual costs of $5.8 billion, while in Canada, it reaches $1.16 billion. In Australia, the cost of violence against women and children is estimated to be around $11.38 billion annually. Domestic violence alone results in costs of approximately $32.9 billion in England and Wales. Similarly, violence against nurses also carries an economic burden with research reporting an average hospital cost per violent incident against a nurse to be $94,156, including $78,924 for treatment and $15,232 for indemnity (Speroni et al., 2014). Moreover, the cost associated with nurse turnover is estimated between $21,514 and $88,000 in the United States of America, resulting in the average hospital losing millions (Bae, 2022).

In examining the issue of violence against nurses, it is crucial to situate it within the broader context of societal violence. Throughout Europe and many other regions, there has been a concerning increase in various forms of violence, including femicide, gun violence, and rising murder rates. In 2021, 3.9 per one million women were killed by family members or intimate partners, while in comparison male victims were 1.8 per one million men (Eurostat, 2021). The UN warns that if homicide rates continue to increase at the current rate of 4%, then Sustainable Development Goal 16 on significantly reducing all forms of violence will not be met by 2030 (UN, 2020). In many societies worldwide, particularly in low-income countries, women endure the greatest weight of fatal victimization due to enduring gender inequality, dependency, and the persistence of misogynistic beliefs.

These alarming social trends cannot be dissociated from the safety and well-being of healthcare professionals, particularly nurses who predominantly bear the brunt of such violence. To comprehend the complexities of this issue fully, it is imperative to acknowledge the pervasive nature of violence and its impact on healthcare organizations. By recognizing violence against nurses as a workplace safety concern rooted in the failure to address violence at the organizational level, we point to a need for legislation and policies that uphold minimum standards for safe working environments. Moreover, it is essential to align such policies with global efforts aimed at reducing violence and crime, as outlined in the UN Sustainable Development Goals. By embracing a broader perspective and understanding the interplay between violence within the healthcare setting and the wider societal context, we point to a need for a more comprehensive understanding of the issue.

Based on the above-mentioned issues concerning violence towards nurses, the aim of this article was to report on a pan-European mapping exercise on challenges and solutions related to violence against nurses within the EU and Europe.

## Method

### Design

A cross-sectional, online survey design was used for this study.

### Sample

Data was collected from members of the European Federation of Nurses (EFN), representing National Nurses’ Associations from countries across Europe.

### Data Collection

An online questionnaire was developed based on a literature review and expert consultation. Face validity was achieved through a group of experts. The questionnaire consisted of closed and open questions (Table 1). Respondents provided written input via an online survey (April 2021) and oral input at the EFN General Assembly *Tour de Table*. At each General Assembly of the EFN, the *Tour de Table* allows EFN members to share information and best practices on a specific topic that should be put higher on the EU political agenda. Besides, key issues and developments of national importance are discussed.

**Table 1.** Survey Questions.

**1.** Are nurses in your country experiencing any type of gender-based violence or discrimination at their workplace?
**2.** Are there initiatives and/or projects put in place to tackle this problem of violence against nurses in your country and/or health settings?
**3.** Are there training programs for nurses on the risks of violence and how to prevent, identify and cope with it in your country and/or health settings?
**4.** Are the employers and/or the national government in your country putting in place policies and/or initiatives for the reduction of violence against nurses at the workplace?
**5.** Please provide any additional information, comments, or challenges nurses are experiencing in your country in terms of gender-based violence or discrimination.

### Data Analysis

Quantitative responses were summarized using descriptive statistics, including counts, percentages, and tabulation. Open questions were analyzed using thematic analysis and narrative synthesis, which involved grouping responses under inclusive headings and reporting these under key areas of political and human resource policy relevance.

### Ethical Considerations

A requirement for formal ethical review was waived given the focus on nursing organizations rather than the individual. The anonymity of participation was assured in the analysis and published accounts. Participation was voluntary, with the information provided via a recruitment email circulated among the EFN members. Submission of the online questionnaire implied consent.

## Results

The present study reports on input from 28 National Nurses’ Associations, representing a response rate of 80% of EFN Members. To simplify the presentation, the number and percentage of countries that responded are provided along with notable examples in brackets. Further information on each country can be obtained from the EFN by visiting www.efn.eu.

### Nurses’ Experiences of Violence

The majority (27/28, 95%) of respondents confirmed that violence against nurses is a significant concern (e.g., Albania, Croatia, Czech Republic, Denmark, Finland). Most drew their evidence from national studies. However, 19% reported a lack of national studies (e.g., Belgium, Estonia, France, Portugal, and Slovakia), and a further 8% noted difficulty establishing a gender dimension to violence (e.g., Cyprus and Spain).

Different kinds of violence were reported, including verbal and physical attacks (e.g., hitting, kicking, biting) and unwelcome sexual attention and harassment. For example, Denmark, Portugal, and the United Kingdom pointed to up to 30% of nurses being sexually harassed in the workplace. The risk of violence for health professions overall can be as high as 80% (e.g., Croatia and Germany). Women appear to be especially vulnerable with twice the risk of being victims of violence (e.g., Estonia and Sweden).

In addition, three alarming trends were revealed from members’ reports: under-reporting of violent incidents, perpetrators of violence, and effects on nurse retention. First, there are serious concerns about under-reporting violent incidents due to fear of victimization and overall employer discouragement; this holds especially true for female nurses (e.g., Bulgaria, Spain, Ireland, Croatia, Albania, and Portugal). Nurse respondents did not perceive reporting would make any difference.

Second, perpetrators of violence against nurses include patients, families, and other health professionals. Up to 41% of nurses report abuse from other professionals (e.g., Germany) with much of this abuse originating from physicians (e.g., Slovenia, Cyprus, and the Czech Republic). Participants perceived gender-based violence against nurses to be associated with their weak position in the hierarchy, subordination by other professions, and lack of social and political power.

Third, violence against nurses has a potentially negative effect on nurse retention. Representative associations reported that nurses reduce their working hours or opt for part-time work as a consequence of violence, with reported estimates of increasing up to 70% the likelihood of leaving the profession (e.g., Switzerland and Ireland).

### Initiatives to Prevent and Respond to Violence Against Nurses

Most countries (75%) have training programs directed at frontline health professionals and their managers (e.g., Portugal, Slovenia, Spain, Sweden, and Switzerland). In some countries, there are generic safety-related programs, but not necessarily specific to violence against nurses (e.g., Croatia, Cyprus, and Ireland). While some of these programs are coordinated at the national level (e.g., Portugal, Bulgaria, Italy, and France), others are developed locally at the regional, health authority, or hospital level.

For example, in Portugal, a national program—to raise awareness through campaigns on early detection of risks and antecedents to violence—has been in place since 2019. Similarly, in Italy and France, there is continued professional development (CPD) training offered at the national level; in Italy, there is also a national day for education on and prevention of violence. Moreover, in some countries, the issue of violence against nurses is now included in the core national nursing curriculum (e.g., Finland and Switzerland), further demonstrating the widespread concern among the nursing community.

In those countries where training is not coordinated at a national level, there are reports of local training initiatives often coordinated by the National Nurses Association. For example, in Slovenia, much of the training is provided by the management of local health settings, often supported by the Nurses and Midwives Association of Slovenia in collaboration with local trade unions and other non-governmental organizations. Attendance at such training opportunities is often at the individual nurses’ initiative and not necessarily encouraged by employing organizations (e.g., Lithuania).

### Policies and Legislation Related to Workplace Violence

A promising trend relates to the availability of policies and legislation to counter violence against nurses. Specifically, 77% of respondents indicated policies and/or legislation already in place at the local, regional, or national level (e.g., Denmark, Estonia, Finland, France, Germany). The remaining countries noted a lack of such policies (e.g., Ireland, Slovakia, and Switzerland) or availability of related but not specific policies on violence against nurses (e.g., Iceland, Lithuania, and the United Kingdom).

Many respondents reported the availability of such policies and/or legislation to be recent, with many implemented in the last 5 years. For example, in 2020, new legislation was passed in Sweden and Italy against violence in the healthcare sector. In Portugal, two resolutions have been approved by parliament to prevent violence against health professionals; in Croatia, legislation is in place to protect healthcare workers with implications for up to 5 years imprisonment for those committing violent acts against them. However, despite existing legislation, some EFN Members remain concerned and caution that the challenge does not lie in the lack of legislation but rather in the lack of enforcement. For example, in Switzerland, due to underfunding and personnel shortage, employers claim that they cannot afford to ensure their personnel's working conditions remain in line with the legal requirements.

It is also worth highlighting that in some countries (e.g., Spain and the Czech Republic), there are national observatories set up to monitor incidences of violence against healthcare professionals. Unfortunately, this is not the case across Europe, thus limiting precise estimates of violent incidents at the EU level. Finally, in Spain, legislation identifies nurses as public authority figures, with any violent incidents against them treated in the same way as violence against police officers.

### Best Practices Shared by EFN Members

Examples of best practices were identified. For example, the Czech Republic launched a comprehensive course on violence prevention and self-protection for nurses. Estonia also launched courses led by psychologists, which cover topics such as professional communication, the importance of self-value and self-awareness, solving conflicts, and developing discussion skills. Finland introduced the “Prevention of threatening and dangerous situations, and identification and management of risks” as part of the competencies of occupational safety regulations.

Moreover, France defined a closer link between hospitals and the police to improve security in public and private hospitals. Specifically, a contact person is designated for each hospital; hospital staff who are victims of violence are supported to file a complaint; active monitoring of emergency departments; and a security diagnosis made by the police for any health structure or health professional practice. Similarly, in Greece, the presence of security staff in the ICU and other high-intensity departments has been institutionalized to protect staff from visitors and patients. In each hospital, specific policies, measures, and actions are activated to reduce violence among the staff.

In Italy, counseling centers and psychological aid are offered to health professionals who have been victims of violence. A National Observatory for violence against healthcare staff promotes studies for reducing health professionals’ exposure to risk factors; monitors the implementation of safety measures, including video-surveillance tools; and promotes best practices and specific courses for health professionals. In Spain, two tools are used to tackle violence against nurses: a phone number (116) that leaves no trace of the call log to avoid retaliation; and a mobile phone application connected to the police called AlertCop.

Finally, in the United Kingdom, the National Health Service (NHS), working with the police and Crown Prosecution Service, helps victims give evidence and get prosecutions most quickly and efficiently. The Care Quality Commission (CQC) scrutinizes violence as part of its inspection regime and identifies NHS hospitals needing further support. There is also improved training for staff to deal with violence, including circumstances involving patients with dementia or mental illness, and prompt mental health support for staff who have been victims of violence.

## Discussion

As the responses to the current survey reveal, the issue of violence against nurses has grown into epidemic proportions. The scale of the issue is significant, with many nurses across Europe being subject to violent incidences regularly. These results are, unfortunately, not surprising. The nature of nurses’ work makes them vulnerable to physical workplace violence and verbal abuse to the extent that evidence has confirmed how nursing professionals have accepted violent attacks as an integral part of their job (Dafny & Beccaria, 2020; Koritsas et al., 2007).

It is known that violent incidences are more common in mental health and emergency departments, as nurses must confront a particular segment of patients with severe conditions and often express unpredictable behaviors (Caruso et al., 2021). Perpetrators are often socially justified because of their illness, and nurses are expected to understand their abusive behavior, forgive them, and not complain. However, it is not limited to specific hospital departments.

Based on literature reviews over the past decade, many violent cases against nurses can be summarized as being the consequence of organizational deficiencies—which increases work pressure and predispose nurses to abuse by others in general—(Arnetz et al., 2018; Gümüşsoy et al., 2021; Khan et al., 2021; Zhang, Wang et al., 2021), a manifestation of power abuses perpetrated by colleagues from higher ranks (Beale & Hoel, 2011; Giménez Lozano et al., 2021), or the expression of a general discreditation of the profession combined with the persistent discrimination against women in the workplace (FRA, 2021; Petrogloou, 2019; Rechel et al., 2006).

### Protecting Nurses Against Violence

The causes of violence are often multifaceted, and solutions to preventing and managing violence must also reflect that complexity. There are implications across the macro, meso, and micro levels.

At the macro level, the level of a national initiative, existing legislation should be monitored for its implementation and successful enforcement. Unless national processes are in place to enact existing legislation, the situation is unlikely to improve. The examples from some countries establishing national observatories for violence against nurses could prove powerful to develop reliable data about the scale of the problem and enable the identification of workplaces needing closer attention and support.

At the meso level, the level of healthcare organizations, sustained efforts should be in place to deter violence against nurses and support nurses when subject to violent incidents. Importantly, clear messaging should be available that encourages nurses to report violent incidents without fear of retaliation, humiliation, or victimization. Unless reporting violence becomes a routine practice among nurses, perpetrators will continue their violent acts without fear of prosecution.

At the micro level, the level of everyday practice, all health professionals should unite behind the common goal of reducing and preventing violence by supporting each other. It is worrying that over a third of nurses in the current survey reported other health professionals, particularly physicians, as the perpetrators of violent acts against them.

For the above initiatives to be successful, the wider public also needs to be educated about the damaging effects of violent behavior on nurses and the quality and safety of the care they or their family members receive. A public awareness campaign could help raise the profile of and respect for nursing. Especially considering nurses’ significant contribution during the COVID-19 pandemic (De Raeve et al., 2021). Without effective action, and as the EFN Members’ reports indicate, healthcare systems across Europe may find themselves in a downward spiral in which violence discourages and drives nurses away from healthcare, which in turn compromises the resilience of health systems triggering public anxiety and frustration that lead to even more acts of violence.

Considering the data shown here, countries across Europe should mobilize to provide better care for all nurses who are victims of violence and abuse. The fact that most nurses are women is inescapable; therefore, initiatives to support women and nurses must be closely aligned. Such initiatives to support women should consider severe prison sentences, legal sanctions against perpetrators of violence, and better training for police officers and legal and health professionals to ensure fair protection for victims. There are some positive steps in this direction which should be supported. For example, the EU Victims’ Rights Directive (EC 2012/29/EU) and strategy (EC COM/2020/258) present a clear strategy to offer better protection and empowerment of women. In this Directive, the topics covered include training to identify and help victims, support tools for women such as shelters, and abuse reporting mechanisms.

Moreover, the new Victims’ Rights Platform^1^ aims to ensure women's rights and better protection from violence and harassment. EU action is required to create social conditions to deal with the aggressive and violent situation nurses face. It is vital that nurses feel free to express themselves and report violent incidents at work in national databases.

### Policy Recommendations

Workplace violence is a complex problem, requiring a set of interventions at different levels. Given different frameworks to classify interventions, we suggest a binary perspective: an enabling external environment and promoting internal capacity.

Workplace violence is an occupational problem that can be resolved through a set of interventions at different levels of resolution. Given the wide amount of literature suggesting different frameworks to classify the scope of interventions, a binary perspective with which to view the set of potentially effective interventions can be considered. Policy options need to consider the complementary role of creating an enabling *external environm*ent (i.e., law enforcement, legislative changes, and organizational support/resources) and aiming at promoting *internal capacity* within the profession (i.e., training, empowerment, and voicing channels for nurses).

***External Environment***. An effective way to conceive evidence-based interventions against workplace violence is through the lenses of three major dimensions: policy updates, procedure enhancements, and education (Adams, 2018). Policy updates are often the most effective but also the slowest to implement. Legislative changes constitute the broader, more encompassing dimension. Yet, legislative changes also depend on the maturity of each EU Member State's legal framework, as legislations proceed at different paces and with varying cuts of scope. Furthermore, there is the added challenge of different sociocultural attitudes on what constitutes workplace violence and harassment, which is a significant hindrance in defining what constitutes violence in the first place (Karatuna et al., 2020).

Eurofound conducted a study mapping the level of development of different types of public preventive policies for tackling workplace violence across the EU Member States (Giaccone et al., 2015). They found that EU national policy interventions could be classified according to different approaches: recommendation to follow general prevention measures against psychosocial risk, promotion of non-binding measures to encourage employers’ actions, and the imposition of actual legal obligations on the employer to intervene beyond the scope of risk preventions. Moreover, five different national-level governance patterns for prevention policies were identified, ranging from decentralized activities to coordinated governmental initiatives with social partners. Given the fragmented landscape, the acceleration of national legislative changes could be effectively achieved through EU institutions exerting pressure on Member States, compelling them to adhere to a progressively comprehensive set of specific measures addressing workplace violence.

Increased security fits in the broader scope of improving the organizational context of healthcare facilities. Other similar changes include improvements in the workplace and managerial procedures, such as restricting the number of relatives entering wards, improving the doctor-patient ratio, or introducing a method to “flag” patients in the electronic medical records to alert staff of potential risks (Kumari et al., 2020). Intervening at the level of organizational processes of work and healthcare services can yield a significant impact as well.

Evidence suggests that multi-component interventions can produce greater effects, such as introducing workplace violence reporting systems and structured education programs (Bordignon & Monteiro, 2021; Somani et al., 2021). Availability of funding and management of existing organizational resources play a role in securing the workplace; for example, the architectural design of emergency departments has been shown to influence the incidence of violence (Lenaghan et al., 2018). The management of human resources is a powerful domain of intervention, at the cross-section between the external environment and internal capacity. Human resources practices can design and implement ways to encourage job autonomy for nurses and empower their role by including them in the decision-making processes of their work environment (Pariona-Cabrera et al., 2020; Rajabi et al., 2020).

***Internal Capacity.*** One of the most transformative tools for promoting internal capacity within the profession is enhancing educational opportunities for nurses and doctors to empower and instill awareness of best practices among healthcare professionals. Structured education implies setting up periodic training programs that inform nurses on how to respond to workplace violence by patients and how to mature effective interpersonal skills in clinical settings, along with equipping supervisors with the leadership skills to be aware of workplace dynamics and provide the support that disrupts negative social behaviors (Janzen et al., 2022; Naveen Kumar et al., 2020; Shier et al., 2021).

Training is one of the most effective tools for reducing violence incidence (Vijayalakshmi et al., 2021). Apart from training, the public has a central role in workplace violence as well: influencing public attitudes can significantly reduce incidences of violence (Lian & Dong, 2021; Warshawski et al., 2021). The public is generally sympathetic toward nurses subject to violence (Tung et al., 2021). Therefore, leveraging the consensus of the public by bringing awareness to the problem of workplace violence can be a leveraging tool for an advocacy organization to place the topic higher in the national agenda of priorities and to advance strategies aimed at ameliorating the public perception and reputation surrounding the nursing profession.

### Limitations

These results should be interpreted in the context of limitations inherent to survey designs. Our results represent a snapshot of data available in March 2021, collected in the middle of the COVID-19 pandemic, so we cannot track progress longitudinally; however, the current article was considered for the currency of data by the EFN members, so we remain confident in the representativeness of our results. Participation in the survey was voluntary, which may skew results like in all surveys; the 80% response rate somehow contains this risk. Finally, the face and content validity of the questionnaire was assured through EFN expertise. However, the risk of missing some potentially relevant items remains; we sought to minimize this risk by including space for written input.

## Conclusion

Violence against nurses is ultimately an expression of a broader problem rooted in organizational inefficiency and the weak application of legislation to maintain minimum standards of safe work environments. What emerged from the testimonies of European nurses in the current survey is, in part, the consequence of weak law enforcement, combined with an inadequate coherent EU-wide legislation targeting workplace violence in healthcare. Nurses would benefit from more institutional support through dedicated funding aimed at targeted interventions, more legislative commitment to ratify policies against discrimination, and the opportunity to voice their concerns to the appropriate policymakers with the ability to bring significant change to existing conditions.

Violence and harassment against nurses are not new. Still, they remain unacceptable, especially given the enormous negative impact on nurses’ psychological and physical well-being, job motivation and turnover, quality of care, and risk to patient safety. It is crucial that nurses, as a predominantly female workforce, are enabled to have a strong voice in the design of health policies. They are ideally positioned to lead and support such developments and, in doing so, ensure reform that addresses the long-standing inequality between women and men, both as providers and as recipients of care.

European and international institutions should look at the achievements to date alongside current risks and discuss the next steps forward in strategic cooperation between EU institutions and Member States, International Organizations, NGOs, and researchers in combatting gender-based violence, towards nurses in particular. Nurses must be protected and supported through the development of policies, initiatives, and legislation at the national and European levels. Given the severity of the situation, inaction could lead to irreplaceable damage to the nursing workforce, compounding pressures resulting from the COVID-19 pandemic. Ultimately, if left untreated, this situation could drive nurses out of the profession.

## Notes

1. European Commission Victims’ Rights Platform. Online: https://ec.europa.eu/info/policies/justice-and-fundamental-rights/criminal-justice/protecting-victims-rights/victims-rights-platform_en
